# Exocyst inactivation in urothelial cells disrupts autophagy and activates non-canonical NF-κB signaling

**DOI:** 10.1242/dmm.049785

**Published:** 2022-10-12

**Authors:** Michael A. Ortega, Ross K. Villiger, Malia Harrison-Chau, Suzanna Lieu, Kadee-Kalia Tamashiro, Amanda J. Lee, Brent A. Fujimoto, Geetika Y. Patwardhan, Joshua Kepler, Ben Fogelgren

**Affiliations:** ^1^ Center for Biomedical Research at The Queen's Medical Center, Honolulu, Hawaii 96813, USA; ^2^ Department of Anatomy, Biochemistry and Physiology, John A. Burns School of Medicine, University of Hawaiʿi at Manoa, Honolulu, Hawaii 96813, USA; ^3^ Math and Sciences Department, Kapiolani Community College, Honolulu, Hawaii 96816, USA

**Keywords:** Autophagy, Exocyst, Fn14, NF-κB signaling pathway, Urothelium

## Abstract

Ureter obstruction is a highly prevalent event during embryonic development and is a major cause of pediatric kidney disease. We have previously reported that ureteric bud-specific ablation of the gene expressing the exocyst subunit EXOC5 in late murine gestation results in failure of urothelial stratification, cell death and complete ureter obstruction. However, the mechanistic connection between disrupted exocyst activity, urothelial cell death and subsequent ureter obstruction was unclear. Here, we report that inhibited urothelial stratification does not drive cell death during ureter development. Instead, we demonstrate that the exocyst plays a critical role in autophagy in urothelial cells, and that disruption of autophagy activates a urothelial NF-κB stress response. Impaired autophagy first provokes canonical NF-κB activity, which is progressively followed by increasing levels of non-canonical NF-κB activity and cell death if the stress remains unresolved. Furthermore, we demonstrate that ureter obstructions can be completely rescued in *Exoc5* conditional knockout mice by administering a single dose of the pan-caspase inhibitor z-VAD-FMK at embryonic day 16.5 prior to urothelial cell death. Taken together, ablation of *Exoc5* disrupts autophagic stress response and activates progressive NF-κB signaling, which promotes obstructive uropathy.

## Teaser

**Summary:** Targeted *Exoc5* deletion in embryonic ureter urothelial progenitor cells impairs autophagy, causing Fn14 activation and urothelial cell death, with a lethal congenital obstructive uropathy rescuable by administration of a caspase inhibitor.

## INTRODUCTION

Ureter obstruction during fetal development is a common cause of children being born with congenital anomalies of the kidney and urinary tract (Chua et al., 2019; Johansen et al., 2021; Verbitsky et al., 2019). The most prevalent site of obstruction occurs at the ureteropelvic junction (UPJ), where the renal pelvis transitions into the upper ureter, resulting in restricted urine flow that can cause lasting kidney damage (Roth et al., 2002). Whether the obstruction resolves naturally or is surgically corrected, as many as 70% of patients with congenital obstructive uropathy (COU) develop a gradual loss of kidney function and progress to end-stage renal disease by the age of 20 (Chevalier et al., 2010; Craven et al., 2007; Mesrobian and Mirza, 2012; Miklovicova et al., 2008). However, there remains a limited understanding of the mechanisms that govern the embryonic onset of COU.

Several mouse models have implicated urothelial abnormalities as a major driver behind ureter obstructions (Jackson et al., 2020). The urothelium is a specialized stratified epithelium that functions as a urine permeability barrier along the upper urinary tract and bladder. We have previously reported that conditional knockout of the gene *Exoc5*, which encodes an exocyst subunit, in ureteric bud cells disrupts the urothelial stratification process in embryonic ureters, which subsequently triggers cell death between embryonic day (E) 16.5 and E17.5 (Fogelgren et al., 2015). The *Exoc5* conditional knockout (*Exoc5* CKO) was achieved using the Ksp-cadherin Cre mouse strain (Cre^Ksp^), in which we demonstrated Cre to be active <E12.5, well before the urothelial cell death began, indicating the disruption of stratification might be key to triggering cell death. The wave of cell death in *Exoc5^FL/FL^;Cre^Ksp^* ureter was followed by a wound healing response that causes UPJ lumen obliteration through the activation and expansion of myofibroblasts (Fogelgren et al., 2015; Lee et al., 2016). The resulting phenotype of the *Exoc5^FL/FL^;Cre^Ksp^* (hereafter referred to as *Exoc5* CKO) mouse is similar to intrinsic human congenital UPJ obstructions and is a valuable model for investigating the underlying mechanisms of ureter obstruction.

EXOC5 is a core component of the highly-conserved octameric exocyst protein complex (consisting of EXOC1-8), which mediates the targeting and docking of intracellular vesicles (Ahmed et al., 2018; Heider et al., 2016; Mei et al., 2018; Morin et al., 2010; TerBush et al., 1996). As the exocyst associates with specific vesicles through members of the Rab GTPase family, the exocyst is often classified as a Rab effector complex. The localization and assembly of the exocyst holocomplex is guided in part by members of the Ras superfamily of small GTPases such as RALA and RALB, which regulate distinct biological processes by interacting with different exocyst subunits. As an example, the RALA–EXOC2 complex mediates polarity by trafficking proteins to the basolateral membrane in epithelial cells (Moskalenko et al., 2002), whereas the RALB–EXOC2 complex can directly activate the innate immunity response and restrict apoptosis by engaging the IκB [inhibitor of nuclear factor-κB (NF-κB)] kinase family member TBK1 (Chien et al., 2006; Shipitsin and Feig, 2004). Alternatively, RALB–EXOC8 can promote autophagy by acting as an assembly scaffold for ULK1 and Beclin1–VPS34 during nutrient deprivation (Bodemann et al., 2011; Martin et al., 2014; Singh et al., 2019). As these different interacting combinations can activate distinct responses, a clearer understanding of the role the exocyst complex performs in cellular stress response is necessary (Simicek et al., 2013).

For example, studies in *Drosophila* have revealed that exocyst-mediated autophagy is tissue specific and context dependent (Mohseni et al., 2009; Tracy et al., 2016). Autophagy is an evolutionarily conserved lysosomal degradation process known to be highly active during differentiation and development (Mizushima and Levine, 2010), and is critical for many physiological events, such as responding to cellular stress by maintaining homeostasis through the clearance of damaged organelles and proteins (Kroemer et al., 2010; Tang et al., 2020). Interestingly, although autophagy appears to be dispensable for mammalian ureter and kidney development (Goodall et al., 2016; Gump et al., 2014; Komatsu et al., 2005; Kuma et al., 2004; Nezis et al., 2010; Thorburn et al., 2014), several mouse models utilizing tissue-specific autophagy related gene (ATG) knockout demonstrate that deficiencies in autophagy promote progressive pathology by limiting the ability of the organism to respond to stress (Bechtel et al., 2013; Hartleben et al., 2010; Kim et al., 2012; Takahashi et al., 2012).

Here, we tested whether ureter obstruction in *Exoc5* CKO mice was the result of failed urothelial stratification or occurred because of unresolved urothelial cell death, and whether prevention of urothelial differentiation during ureter development directly caused cell death. Furthermore, we used a ureter explant model to determine whether the fibroproliferative wound healing reaction that obliterates the ureter lumen was activated in the absence of urine. Our data show that autophagy was impaired after urothelial *Exoc5* ablation, which triggered an increasing prevalence of the tumor necrosis factor (TNF) superfamily receptor Fn14 (encoded by *Tnfrsf12a*), a potent activator of the non-canonical NF-κB pathway. Our data demonstrate that inhibiting autophagy in urothelial cells provokes an initial p65 (also known as RelA) canonical NF-κB response, which is progressively followed by a secondary p52 (encoded by *Nfkb2*) non-canonical NF-κB response and then cell death. Lastly, we investigated whether the ureter urothelial cell death was critical to COU in our *Exoc5* CKO mice through rescue experiments using the pan-caspase inhibitor z-VAD-FMK at E16.5. Taken together, our data demonstrate that impaired exocyst-mediated autophagy in urothelial cells activates progressive NF-κB signaling that enhances cell death and promotes the onset of COU.

## RESULTS

### Differentiating urothelial cells initiate cell death independently of failed stratification

**Fig. 1.**
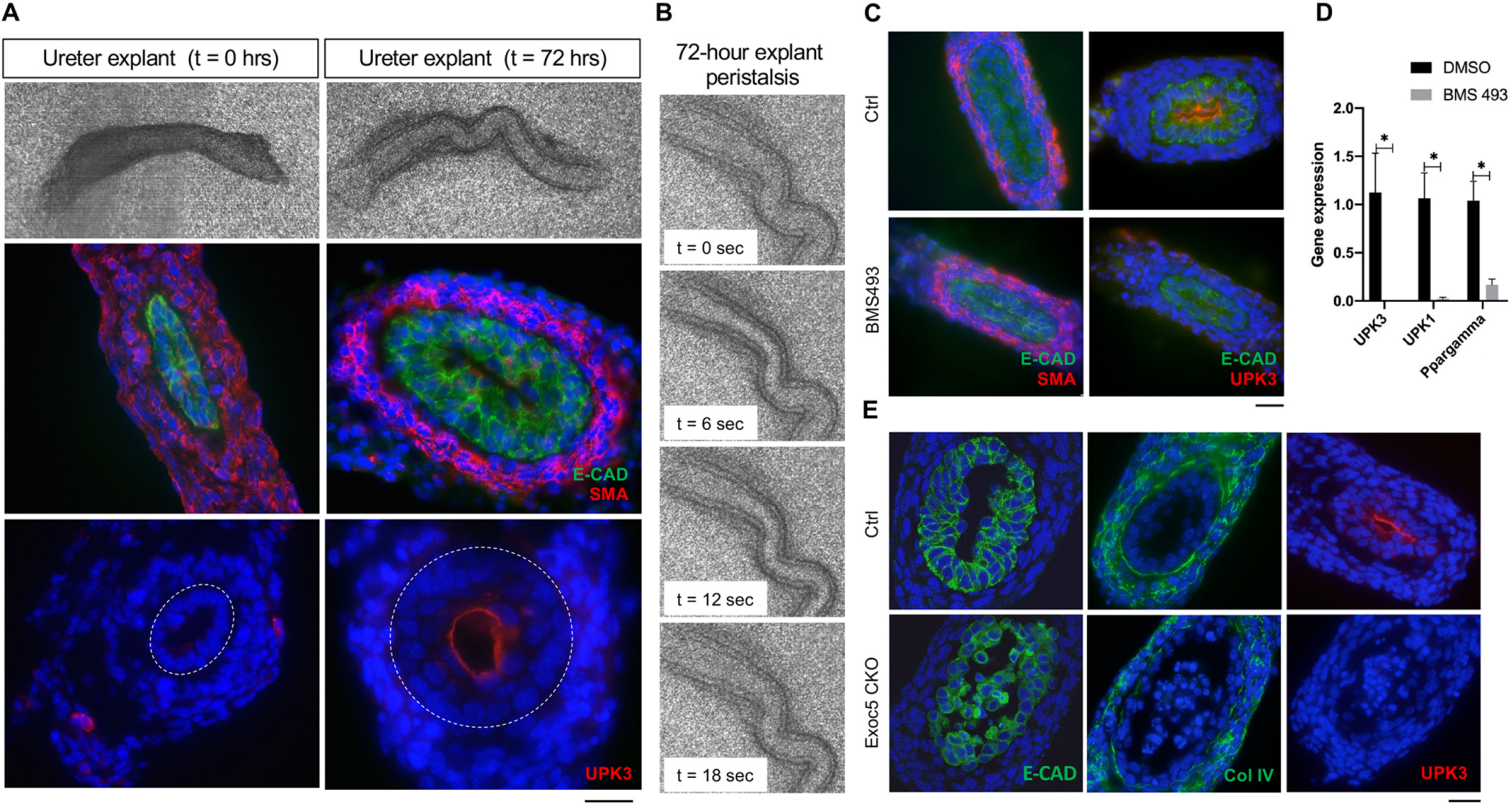
**Urothelial cell death occurs independently of failed stratification.** (A) *Ex vivo* ureter explant culture model resulted in viable *Exoc5^FL/FL^* (control) ureter explants after 72 h**.** E15.5 (*t*=0 h) control ureter explants were cultured for 72 h and are shown. Explants were fixed, cryosectioned and immunostained for E-cadherin (green) and SMA (red), which revealed urothelial stratification and strengthening of the smooth muscle layer. Staining for uroplakin-3 (UPK3) (red) demonstrated successful differentiation of superficial cells *ex vivo*. Dotted circles indicate the border between the urothelium and lamina propria. (B) Time-lapse images of ureter explant peristalsis confirmed functional viability of ureters after 72 h. (C) Wild-type ureter treated *ex vivo* with the retinoic acid inhibitor BMS 493 at 100 μM for 72 h developed a monolayered epithelium and lacked UPK3 expression with no apparent cell death, whereas vehicle-control-treated ureter explants developed a multilayered urothelium as indicated by E-cadherin (green) and UPK3 (red). (D) Real-time qPCR confirmed *Upk3*, uroplakin-1 (*Upk1*) and *Pparg* downregulation in BMS 493-treated ureter explants. Data show the mean±s.d. (two-tailed unpaired *t*-test). *P≤0.05. (E) *Exoc5* CKO *ex vivo* ureter explants cultured for 72 h displayed a disrupted urothelial layer with epithelial cells detaching from the smooth muscle layer and entering the lumen. *Exoc5^FL/FL^* control *ex vivo* ureter cultures showed differentiation of a multilayered urothelium, indicated by E-cadherin (green) and the presence of UPK3 (red). Scale bars: 20 µm.

Abnormal urothelial differentiation has been implicated in mouse models and patients with congenital anomalies of the kidney and urinary tract. To investigate the underlying cause of the obstruction in *Exoc5* CKO embryonic ureters, we developed an *ex vivo* ureter explant culture system. Wild-type C57BL/6J mouse embryonic ureters were collected at E15.5 and cultured for 72 h on semi-permeable supports at the air–liquid interface (Fig. 1A). In this system, the explant urothelium starts as a single epithelial monolayer, as shown by immunostaining for E-cadherin (E-CAD) and smooth muscle actin (SMA) (E15.5, *t*=0 h), and successfully differentiates into a stratified epithelium with uroplakin-3 (UPK3) staining on the luminal surface (E18.5, *t*=72 h). Uroplakins are produced by superficial cells, thus indicating successful urothelial differentiation in an *ex vivo* setting over a time course similar to that of *in vivo* ureter development. After 72 h, the explant ureters showed increased overall growth and continued to have regular peristalsis, indicating the successful maturation of the smooth muscle layer that surrounds the ureters (Fig. 1B).

Upon establishing this *ex vivo* ureter model, we wanted to determine whether failed differentiation was the underlying cause of the urothelial cell death observed in *Exoc5* CKO mice. To test this, we treated E15.5 explant ureters with either vehicle control or the retinoic acid receptor inverse agonist BMS 493 for 72 h, as retinoic acid signaling is known to be necessary for urothelial differentiation (Gandhi et al., 2013). Explant ureters treated with BMS 493 were unable to differentiate into a multilayered epithelium, as shown with E-cadherin/SMA immunohistochemistry (Fig. 1C). The BMS 493-treated ureters also completely lacked uroplakin expression (Fig. 1D); however, no signs of terminal deoxynucleotidyl transferase dUTP nick end labeling (TUNEL)-positive urothelial cell death (Fig. S1) or lumen obstruction were detected. Real-time quantitative PCR (qPCR) analysis of BMS 493-treated samples showed that the level of *Pparg*, a downstream target of retinoic acid signaling, was also strongly downregulated, confirming that BMS 493 successfully disrupted the retinoic acid signaling pathway and urothelial differentiation (Fig. 1D). Furthermore, EXOC5 localization at the apical luminal membrane of urothelial cells (Fig. S1) mirrored that from previous observations using *in vivo Exoc5* CKO ureters (Lee et al., 2016), and it remained unchanged with BMS 493 treatment.

Next, we wanted to use this ureter explant model to determine whether the *Exoc5* CKO urothelial cells still underwent cell death and whether the fibroproliferative obstruction would be induced in the absence of urine flow. For this, we cultured *Exoc5* CKO and littermate control E15.5 explants for 72 h and performed immunohistochemistry. As with the wild-type ureter explants, control ureter explants showed the presence of a normal multilayered urothelium after 72 h, whereas *Exoc5* CKO ureters displayed a disrupted urothelium with urothelial cells sloughing off and entering the luminal space, as seen with E-cadherin (green) (Fig. 1E). As previously described for *in vivo* conditions (Fogelgren et al., 2015; Lee et al., 2016), *Exoc5* CKO ureter explants also showed no uroplakin expression, indicating that the urothelial progenitors failed to differentiate into superficial cells. However, unlike the pathology of *Exoc5* CKO ureters *in vivo*, the distribution of collagen IV indicated an intact basement membrane, with no expansion of mesenchymal cells, suggesting that a fibroproliferative response was not activated as a result of the urothelial cell death (Fig. 1E). Furthermore, we did not find a difference in mesenchymal proliferation as measured by Ki67 (Fig. S2), and only urothelial cells that had sloughed off from the basement membrane were found to be positive for cleaved caspase 3, an apoptosis marker (Fig. S3). Taken together, these results suggest that the epithelial cells comprising the urothelial progenitor monolayer initiate cell death independently of failed differentiation or the presence of urine.

### The NF-κB activator Fn14 is highly upregulated in *Exoc5* CKO ureters

**Fig. 2.**
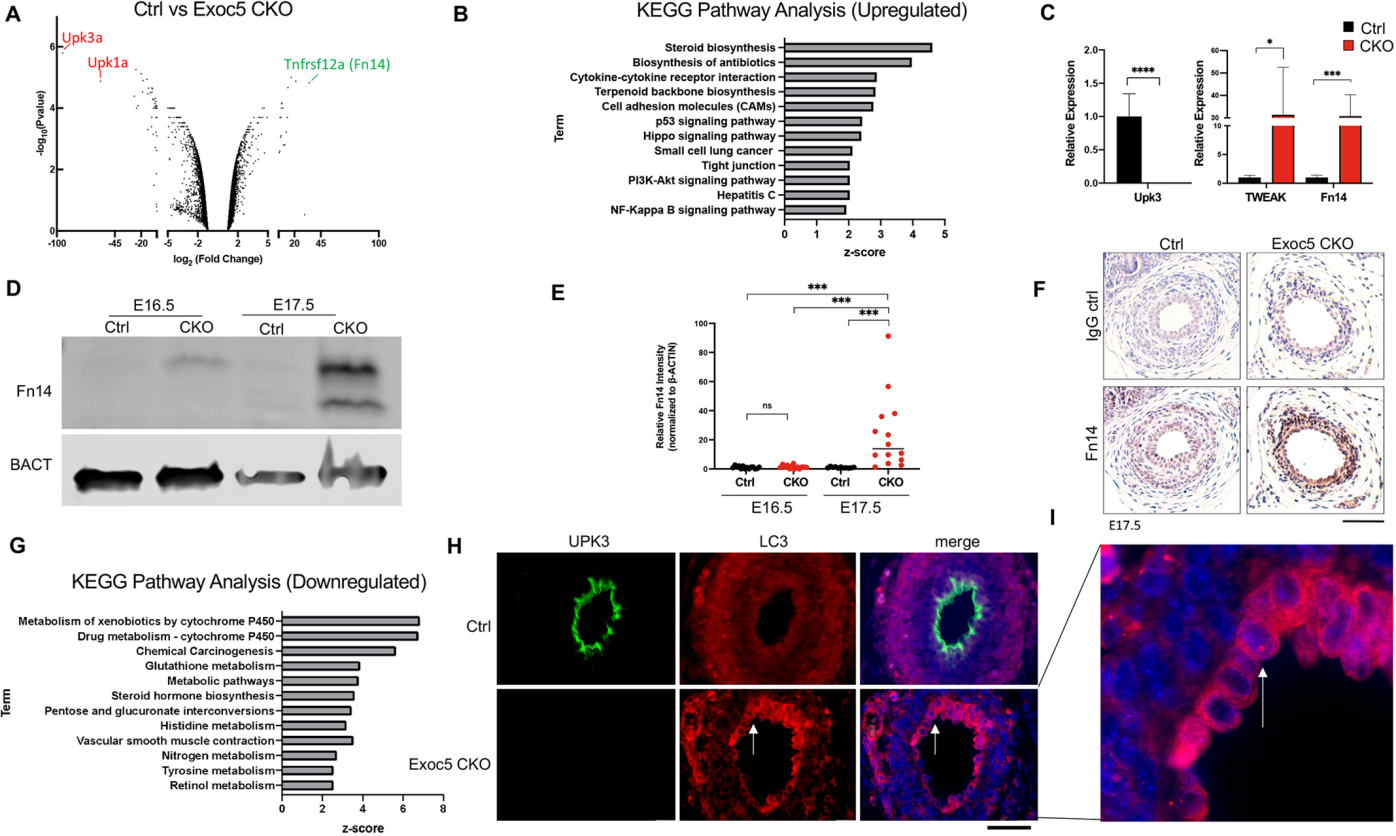
**NF-κB signaling and autophagy are significantly impacted by *Exoc5* ablation in the developing mouse ureter.** (A) Gene-expression profiling of total ureter RNA from control and *Exoc5* CKO mice at E16.5 (*n*=3 for each group). Volcano plot displaying the gene expression analysis shows uroplakin genes as being significantly downregulated (red) and the NF-κB signaling mediator Fn14 gene (*Tnfrsf12a*) as significantly upregulated (green). (B) NF-κB signaling was among the most upregulated pathways identified by KEGG Pathway Analysis. (C) Real-time qPCR confirmed *Upk3* downregulation and *Fn14* upregulation in *Exoc5* CKO E16.5 ureters. In addition, gene expression of the cytokine TWEAK was significantly upregulated along with that of its receptor Fn14 (two-tailed unpaired *t*-test). (D) Western blot analysis of *Exoc5* CKO ureters showed robust expression of Fn14 at E17.5 compared to control ureters or even *Exoc5* at E16.5. β-actin (BACT) was used as a loading control. (E) Quantification of Fn14 detected by western blots in E16.5 control, E16.5 *Exoc5* CKO, E17.5 control and E17.5 *Exoc5* CKO ureters (*n*=14, 14, 11 and 14, respectively), with each dot representing a mouse (two-way ANOVA and Tukey's post hoc test). (F) Immunohistochemistry of E17.5 control and *Exoc5* CKO ureters shows Fn14 localization to the urothelial cells surrounding the ureter lumen. (G) KEGG pathway analysis of the most downregulated pathways indicated broad metabolic disruption. (H) Immunohistochemistry of E17.5 control and *Exoc5* CKO ureters displayed differences in LC3 distribution and the onset of LC3 puncta formation (red, arrow). As expected, uroplakin-3 staining (green) shows that control ureters had stratified by this timepoint, but *Exoc5* CKO ureters had not. (I) Higher magnification of the accumulation of LC3 in the E17.5 *Exoc5* CKO ureter. Data show the mean±s.d. ns, not significant, *P*≥0.05; *P≤0.05; ***P≤0.001; ****P≤0.0001. Scale bars: 20 µm.

We found that only a small number of cells that have sloughed off from the basement membrane of *Exoc5* CKO ureters are TUNEL positive (Fig. S4). To identify molecules potentially involved in the urothelial cell death event in *Exoc5* CKO ureters, we performed gene profiling on RNA samples from microdissected E16.5 ureters using Affymetrix Clariom D GeneChip microarrays. As expected based on our previously reported data (Fogelgren et al., 2015), we measured strongly decreased expression of uroplakin genes in *Exoc5* CKO ureters (Fig. 2A). One of the most upregulated individual genes was *Fn14* (*Tnfrsf12a*), which is a TNF superfamily receptor known to play a role in both canonical and non-canonical NF-κB signaling (Fig. 2A). Fn14 has only one known ligand, a cytokine named tumor necrosis factor-like weak inducer of apoptosis (TWEAK, encoded by *Tnfsf12*), and *Fn14* has been shown to be a stress-response gene in many tissues (Muñoz-García et al., 2006; Nagy et al., 2021; Peng et al., 2018; Unudurthi et al., 2020; Zhao et al., 2007). *Fn14* is often strongly upregulated after cell damage or oncogenic transformation (Johnston et al., 2015); however, there are no published reports of its activity in urothelial cells. We then performed KEGG pathway analysis, which implicated NF-κB signaling among the most upregulated pathways in response to *Exoc5* ablation (Fig. 2B). qPCR validation confirmed the loss of uroplakin expression in these *Exoc5* CKO samples and that expression of *Fn14* and *TWEAK* were both increased by more than 30 fold at E16.5 (Fig. 2C). As Fn14 has a relatively short half-life of ∼74 min (Gurunathan et al., 2014), we performed western blotting on isolated E16.5 and at E17.5 ureters to measure protein levels. Relatively low protein levels of Fn14 were seen at E16.5; however, there was a strong buildup of Fn14 in E17.5 *Exoc5* CKO ureters, indicating that Fn14 levels were elevated during this period of stress (Fig. 2D). The degree of increase in Fn14 levels in E17.5 *Exoc5* CKO ureters varied, but was consistently and significantly higher compared to other genotypes (Fig. 2E). In order to determine where Fn14 was being expressed, we performed immunohistochemistry and identified that Fn14 was strongly upregulated not only in the urothelium, but also in the underlying mesenchymal cells (Fig. 2F). These data demonstrate that the NF-κB signaling activator Fn14 robustly responds to stress induced by *Exoc5* CKO during ureter development.

### LC3 accumulates in *Exoc5* CKO urothelium

KEGG pathway analysis of the most downregulated pathways revealed several metabolic mechanisms as being significantly perturbed following *Exoc5* ablation (Fig. 2G). Given that the exocyst has been implicated in the initiation of autophagic stress response (Bodemann et al., 2011), we reasoned that exocyst-mediated autophagy might be deficient in the urothelium and contributes towards the downregulation of these metabolic pathways. As LC3 is known to accumulate when autophagy is impaired (Runwal et al., 2019), we tested this by performing immunohistochemistry of the LC3 protein in E17.5 *Exoc5* CKO ureters. Although control ureters showed no obvious abnormalities and only low levels of LC3, *Exoc5* CKO ureters displayed a strong accumulation of LC3 in the urothelium; however, the surrounding smooth muscle layer did not show a significant increase (Fig. 2H). It was noted that the *Exoc5* CKO urothelium showed accumulation of LC3 puncta (Fig. 2I, arrow), which is commonly observed when LC3 is not degraded from active autophagy.

### Inhibiting exocyst function causes impaired autophagy in urothelial cells

**Fig. 3.**
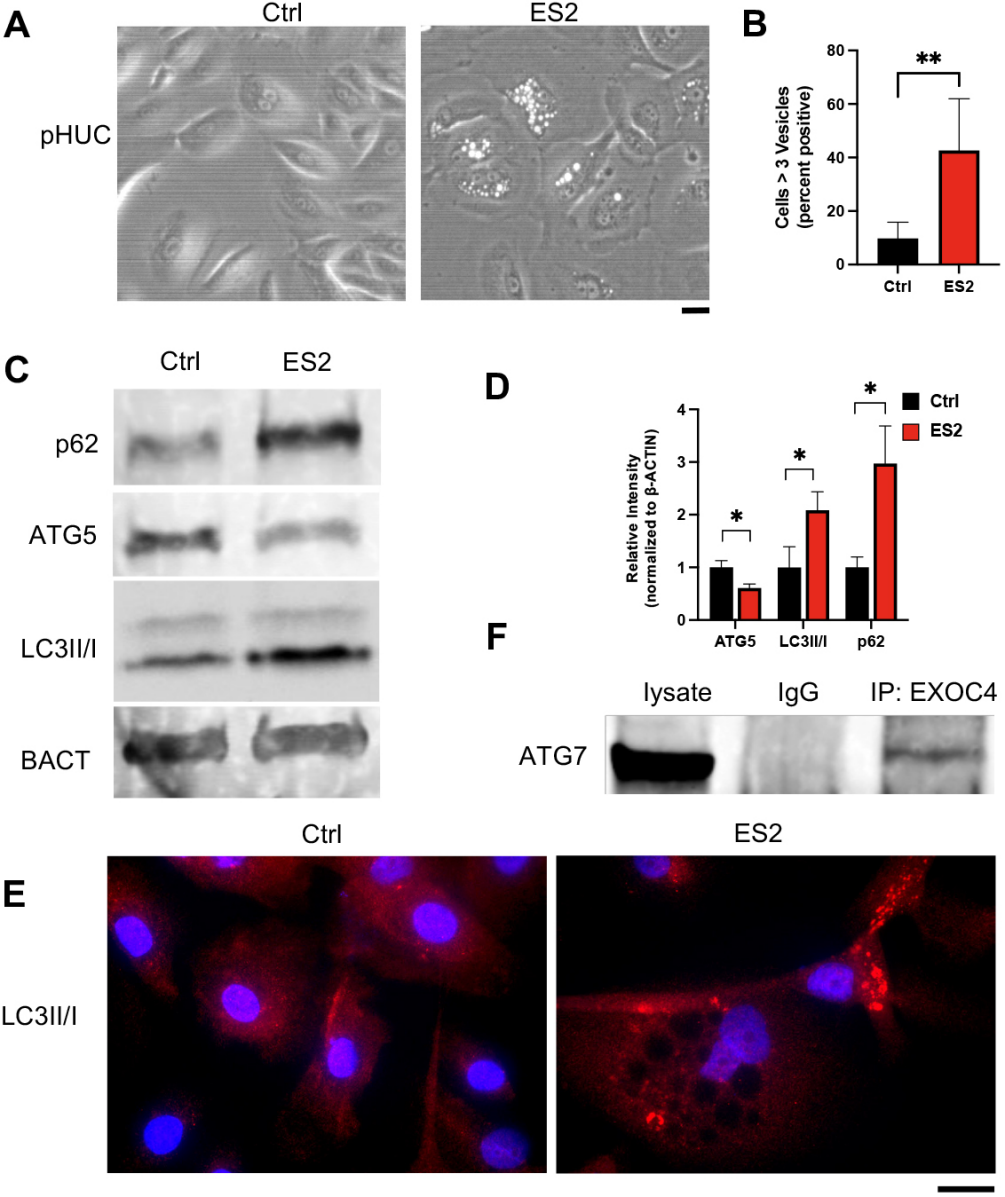
**Inhibiting exocyst function in pHUCs results in decreased autophagy and vesicle accumulation.** (A) Phase-contrast microscopy of 24 h 100 µM endosidin-2 (ES2)-treated pHUCs displays vesicle accumulation. (B) Quantification of control and ES2-treated cells with >3 vesicles (*n*=854 and 778, respectively) shows that ES2-treated samples are more likely to display pHUCs with vesicle accumulation (two-tailed unpaired *t*-test). (C) Western blotting of 24 h ES2-treated pHUCs indicated increased p62 levels and the LC3 II/I ratio, and a reduction in total ATG5 levels. β-actin (BACT) was used as a loading control. (D) Quantification of immunoblotted autophagy markers after 24 h 100 µM ES2 treatment demonstrates a reduction in total ATG5 levels and increase in p62 levels and the LC3II/I ratio (two-tailed unpaired *t*-test). (E) Immunofluorescence detection of LC3II/I in 24 h 100 µM ES2-treated pHUCs displays puncta accumulation. (F) In SV-HUC-1 cells, immunoprecipitation (IP) of EXOC4 successfully pulled down ATG7, indicating a biochemical protein–protein interaction. Data show the mean±s.d. *P≤0.05; **P≤0.01. Scale bars: 20 µm.

Although interactions between the exocyst and ATGs have been previously reported, the functional consequence of perturbing this relationship in urothelial cells remains unclear. Here, we used 100 µM endosidin-2 (ES2) to inhibit exocyst function (Zhang et al., 2016). ES2 treatment of primary human urothelial cells (pHUCs) resulted in progressive vesicle accumulation over 24 h, which was visible by phase-contrast microscopy (Fig. 3A,B). To determine whether ES2 vesicle accumulation was the result of impaired autophagy in pHUCs, we performed western blotting of 24 h ES2-treated pHUCs to measure the protein levels of classic autophagy markers. We measured a decrease in ATG5 levels, and significant increase in the LC3II/I ratio and p62 accumulation after ES2 treatment (Fig. 3C,D). To complement the shift in the LC3II/I ratio, we also observed an increase in the number of LC3 punta (Fig. 3E). To test whether the exocyst and ATGs biochemically associated in human urothelial cells, we performed immunoprecipitation of EXOC4 followed by western blotting for ATG7 using SV-HUC-1 immortalized urothelial cells and observed a positive pulldown (Fig. 3F). These data show that disrupting exocyst trafficking with ES2 treatment disrupts the process of autophagy.

**Fig. 4.**
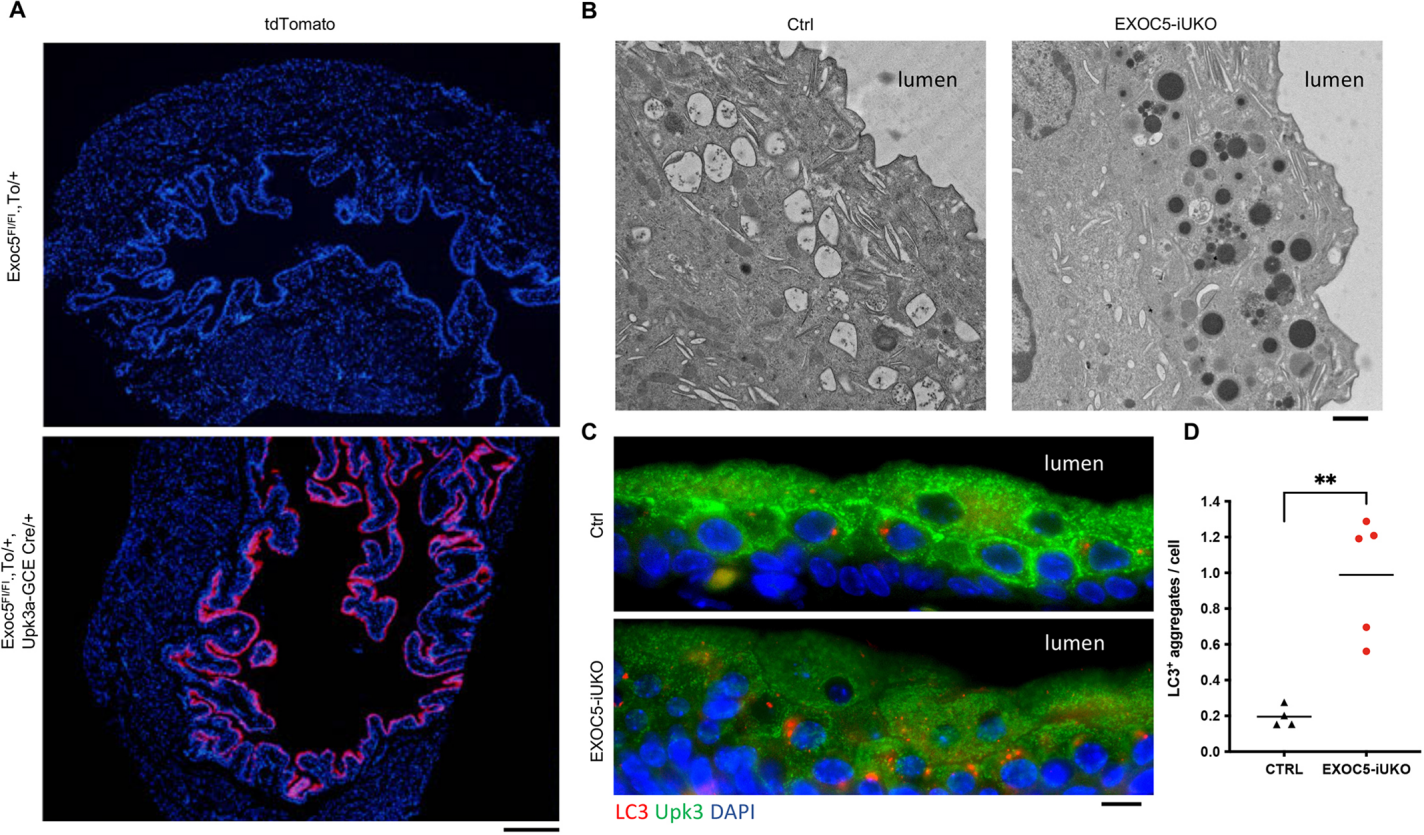
***Exoc5* ablation in adult bladder urothelial cells results in lysosomal accumulation.** (A) Specificity of the Cre reporter system shown by tdTomato (To) activation (red) in superficial urothelial cells. (B) Transmission electron microscopy of bladder sections display lysosomal buildup as marked by electron-dense organelles. (C) Immunohistochemistry of EXOC5-iUKO bladder sections displayed an accumulation of LC3-positive puncta in the superficial urothelial cells. (D) Quantification of LC3-positive aggregates per cell, with each dot representing a mouse. Data show the mean±s.d. (two-tailed unpaired *t*-test). **P≤0.01. Scale bars: 200 μm (A); 1 μm (B); 10 μm (C).

To further demonstrate that inhibiting exocyst function had a detrimental effect on autophagy in urothelial cells, we ablated *Exoc5* in the adult bladder urothelium by crossing *Exoc5^FL/FL^* with *Upk3a-GCE* mice, which express both tamoxifen-activated Cre and GFP under the *Upk3a* promoter (Honeycutt et al., 2015). In parallel, *tdTomato* Cre-reporter mice were crossed with *Upk3a-GCE* mice to assess Cre recombinase activity and specificity after tamoxifen treatments (Fig. 4A). The *Exoc5^FL/FL^;Upk3a-GCE* mice were treated with tamoxifen at 6-8 weeks of age to generate induced urothelial *Exoc5* knockout (*Exoc5-iUKO*) mice, with control mice defined as *Exoc5^FL/FL^* mice treated with an identical regimen of tamoxifen. The *Exoc5-iUKO* mice survived with no gross abnormalities until they were euthanized 4 weeks later for bladder histology and electron microscopy. Scanning electron microscopy on the *Exoc5-iUKO* luminal bladder surface did not reveal any noticeable abnormalities in uroplakin plaque structures, and Haematoxylin and Eosin (H&E) histology was unremarkable. However, transmission electron microscopy on *Exoc5-iUKO* bladder sections revealed a significant buildup of lysosomes as marked by electron dense organelles (Fig. 4B), which was similar to observations found in age-related lysosomal disease models (Truschel et al., 2018). To test for impairments in autophagy in these *Exoc5-iUKO* urothelial cells, we performed immunofluorescent detection of LC3 on bladder sections and observed classical large LC3 puncta formation at a highly increased frequency over those seen in controls (Fig. 4C,D). Taken together, these data indicate that the exocyst biochemically interacts with ATGs in urothelial cells and that inhibiting exocyst function detrimentally affects autophagy in a tissue-specific manner.

### Fn14 is upregulated by inhibiting autophagy in urothelial cells

**Fig. 5.**
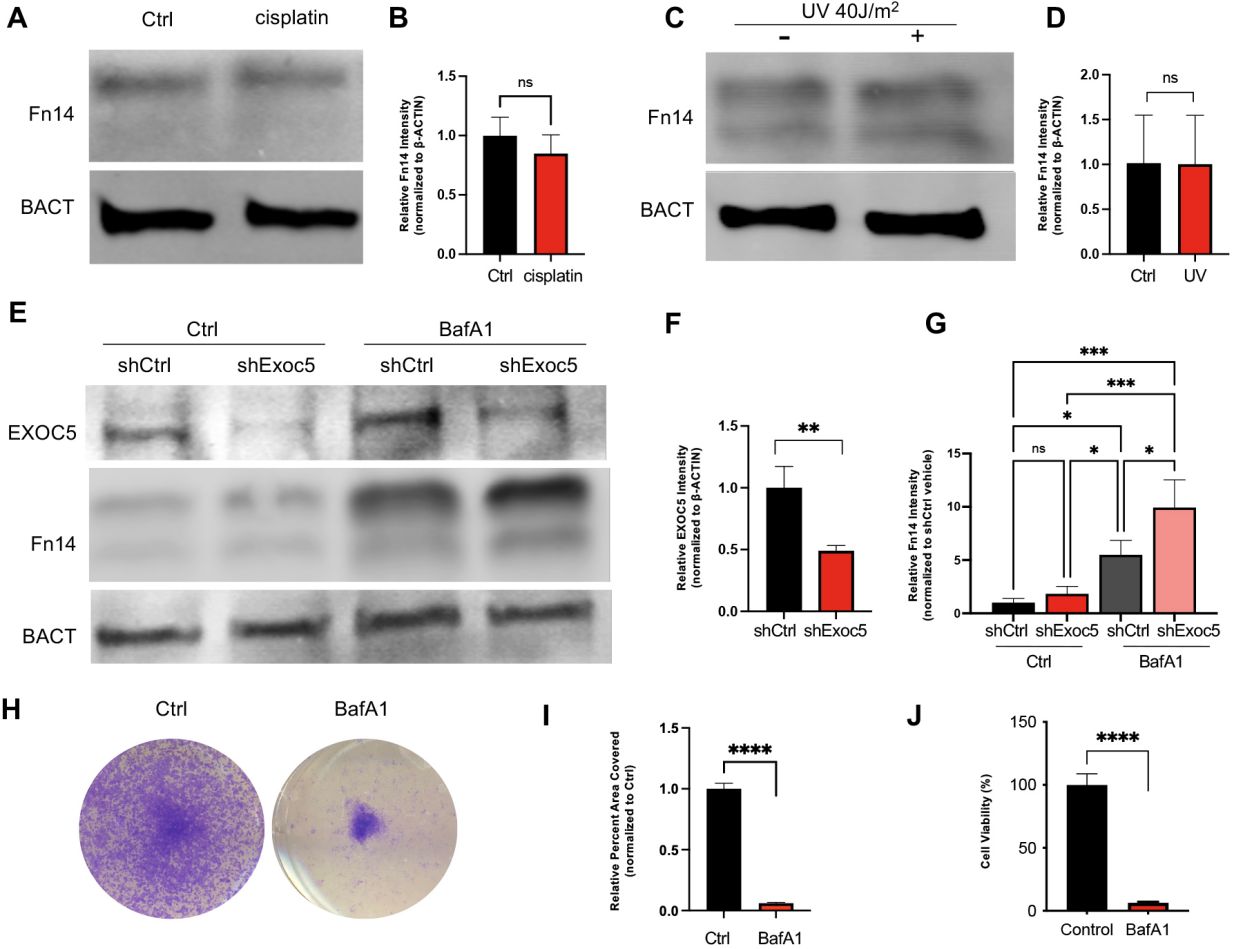
**Autophagy inhibition induces a response by the NF-κB activator Fn14.** (A,B) Western blot analysis of SV-HUC-1 cells treated with 20 µM cisplatin for 4 h to induce DNA damage showed no change in Fn14 levels. (C,D) Western blot analysis of SV-HUC-1 cells that were dosed with ultraviolet light (UV) at 40 J/m^2^ and then left to recover for 6 h showed no increase in Fn14 levels. (E,F) Stably selected shExoc5 SV-HUC-1 cells displayed a synergistic sensitivity in inducing Fn14 response when treated with 200 nM BafA1 to inhibit autophagy for 24 h, compared to shCtrl. β-actin (BACT) was used as a loading control. (G) Western blot quantification of 200 nM BafA1 treatment for 24 h showed increased Fn14 levels in both control and shExoc5 groups and that this effect was the highest in shExoc5+BafA1-treated cells. (H) SV-HUC-1 cells grown for 1 week in the presence of 200 nM BafA1 and stained with Crystal Violet (CV) staining solution showed that inhibiting autophagy significantly impaired growth. (I) Quantification of surface area coverage by CV staining. (J) CellTiter-Glo ATP-based cytotoxicity assay of SV-HUC-1 cells treated with BafA1 demonstrated cell death occurring by 72 h. Data show the mean±s.d. [two-tailed unpaired t-test (B,D,F,I,J); two-way ANOVA and Tukey's post hoc test (G)]. n.s., not significant, *P*≥0.05; *P≤0.05; **P≤0.01; ***P≤0.001; ****P≤0.0001.

We wanted to determine whether there was a causal relationship between impaired autophagy and the non-canonical NF-κB signaling observed in our microarray data. To test this, we investigated whether Fn14 responded to disruptions of autophagy and other forms of stress in SV-HUC-1 cells. First, we treated SV-HUC-1 cells with 20 µM cisplatin for 4 h to assess the effect of cisplatin-induced DNA damage on Fn14 levels and found no significant induction of Fn14 (Fig. 5A,B). Next, we exposed SV-HUC-1 cells to ultraviolet light at 40 J/m^2^ and found no significant Fn14 increase after 6 h of recovery (Fig. 5C,D). These results indicated that under two different forms of DNA damage-induced cell stress, Fn14 was not upregulated in the measured timeframes. Next, to determine whether exocyst depletion impacted the ability of SV-HUC-1 cells to respond to stress, we generated shExoc5 stable knockdowns. We observed by western blotting that treating SV-HUC-1 cells with 200 nM of autophagy inhibitor BafA1 for 24 h caused a significant increase in Fn14 and that this response appeared to be synergistic in shExoc5 knockdown cells (Fig. 5E-G). Treatment of wild-type SV-HUC-1 cells with 200 nM BafA1 over a 5 day period greatly reduced colony formation (Fig. 5H,I) with significantly less cell viability observed after 3 days (Fig. 5J). Interestingly, inhibiting autophagy with either BafA1 or VPS34 inhibitor (VPS34i) both induced vesicle accumulation, which was similar to ES2-treated cells (Fig. S5). These results indicated that inhibiting autophagy in urothelial cells with BafA1 was sufficient to induce Fn14 and promote cell death. These data suggest that EXOC5 knockdown added an additional stress in complement to the direct autophagy inhibition.

### Inhibiting autophagy activates two waves of NF-κB signaling

**Fig. 6.**
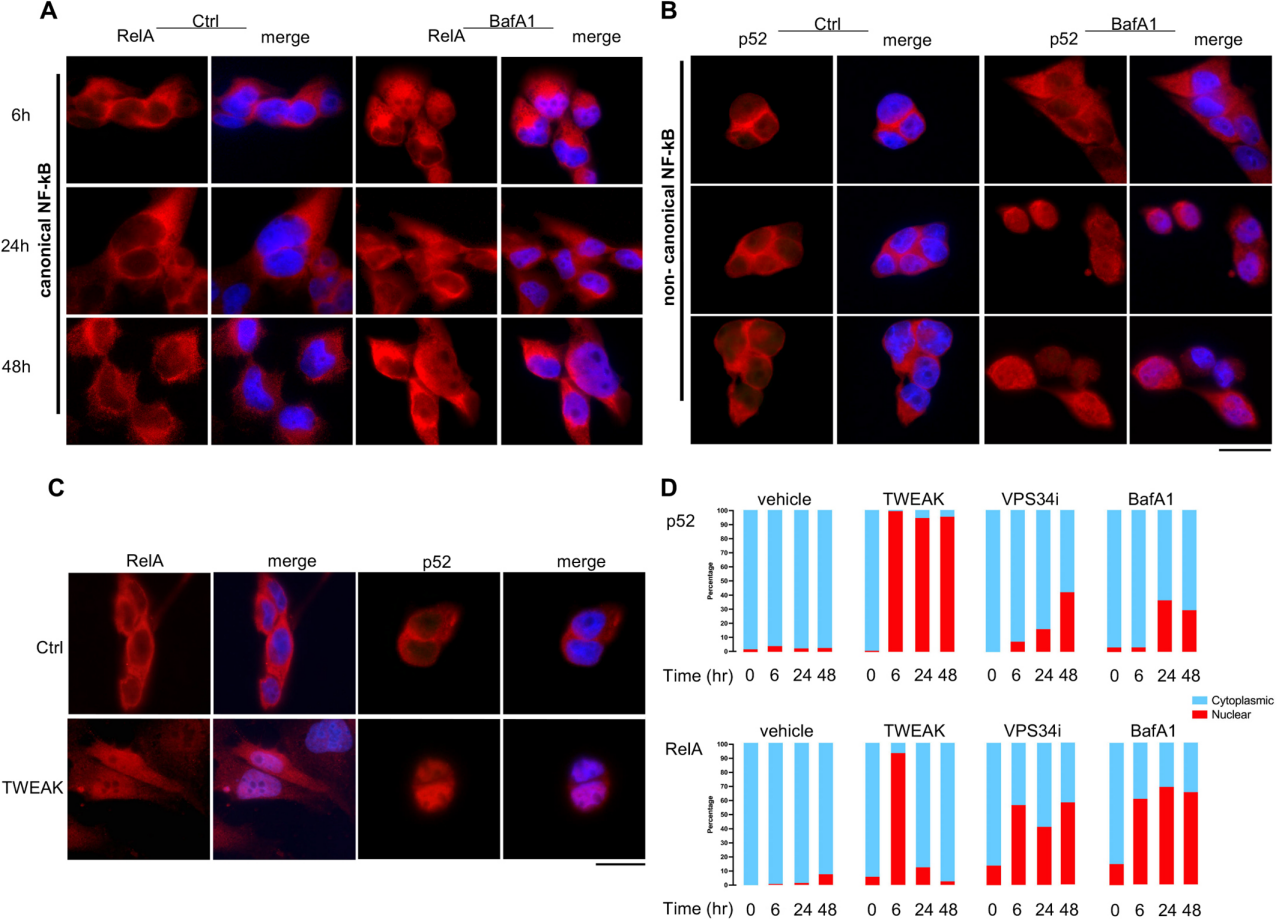
**Inhibiting autophagy in SV-HUC-1 cells activates progressive canonical followed by non-canonical NF-κB signaling.** (A) Immunocytochemistry of 200 nM BafA1-treated SV-HUC-1 cells stained for RelA (red) showed nuclear translocation as early as 6 h, with sustained localization observed at 24 h and 48 h. (B) Immunocytochemistry of 200 nM BafA1-treated SV-HUC-1 cells demonstrated that nuclear translocation of the non-canonical NF-κB signaling constituent p52 (red) was not observed at 6 h but appeared later at 24 h and 48 h. (C) TWEAK-treated SV-HUC-1 cells stained for RelA (red) and p52 (red) demonstrated that both canonical and non-canonical NF-κB signaling were active at 6 h. (D) Quantification of the percentage of cells showing nuclear translocation of RelA and p52 in response to TWEAK, VPS34i or BafA1 treatment at 0, 6, 24 and 48 h. Scale bars: 10 µm.

Next, we wanted to determine whether the NF-κB signaling was responding to cell stress triggered by impaired autophagy in these urothelial cells. First, we performed immunocytochemistry on SV-HUC-1 cells treated with either 200 nM BafA1 or 50 µM VPS34i to determine whether canonical or non-canonical NF-κB signaling pathways were being stimulated with autophagy inhibition. We measured the percentage of cells showing nuclear translocation of RelA as early as 6 h after either BafA1 or VPS34i treatment (Fig. 6A,D; Fig. S5), indicating that canonical NF-κB signaling was active. Nuclear translocation of RelA remained present through 48 h, although varying levels of saturation were observed. To determine whether non-canonical NF-κB signaling was also being stimulated by autophagy inhibition, we performed a similar time course and measured the percentage of cells with p52 nuclear translocation. Although p52 nuclear translocation was not significantly detected at 6 h, we found an increasing prevalence from 24 h to 48 h, nearing the time of significant BafA1-induced cell death (Fig. 6B,D). These data indicate that inhibiting autophagy results in canonical NF-κB activity and that prolonged treatment promotes non-canonical NF-κB activity.

Although TNF superfamily receptors can activate canonical NF-κB signaling, Fn14 is one of the few known receptors to primarily activate non-canonical NF-κB signaling. To confirm that Fn14 signaling was active in SV-HUC-1 cells, we treated the cells with the only known ligand of Fn14, the cytokine TWEAK, and observed a strong increase of both p52 and RelA nuclear translocation by 6 h (Fig. 6C,D). However, only p52 remained activated at significant levels by 24 and 48 h (Fig. 6D). This indicated non-canonical NF-κB signaling can be activated in human urothelial cells through the TWEAK–Fn14 signaling axis, and that although both NF-κB pathways could be activated by TWEAK in urothelial cells, the promotion of non-canonical NF-κB signaling was sustained for longer periods. Taken together, these data indicate that TWEAK and Fn14 activate canonical NF-κB signaling in response to autophagy inhibition as an initial response to stress, but that a second wave of non-canonical NF-κB follows if the insult persists.

### z-VAD-FMK rescues cell death and ureter obstruction in *Exoc5* CKO ureters

**Fig. 7.**
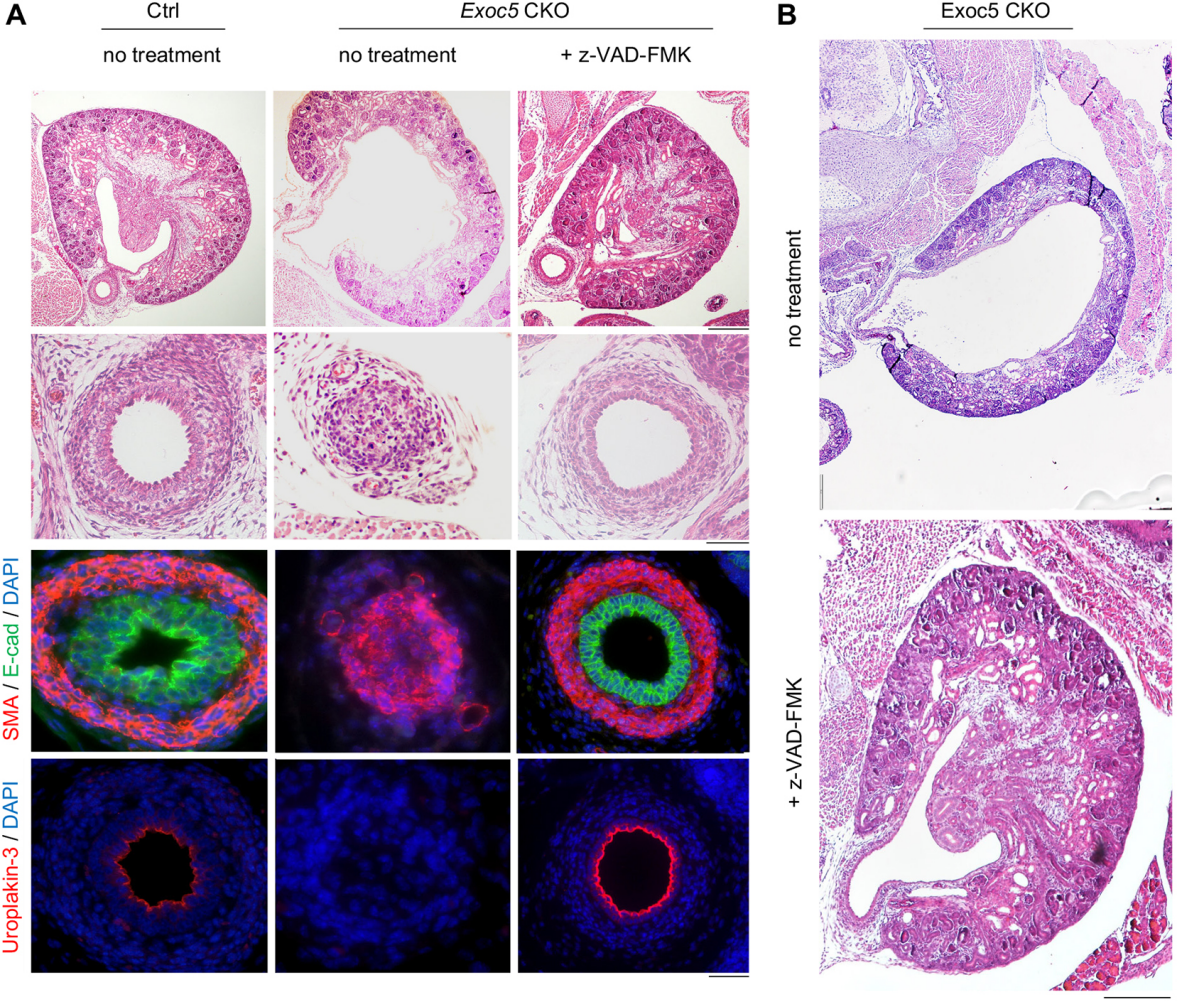
**Single dose of the pan-caspase inhibitor z-VAD-FMK at E16.5 rescues ureter obstruction in *Exoc5* CKO embryos.** (A) H&E staining of E18.5 kidneys and ureters from control and *Exoc5* CKO embryos with no treatment and *Exoc5* CKO embryos with z-VAD-FMK treatment demonstrates rescue of ureter obstruction and the absence of cell death. Immunohistochemistry staining of the same series showed that treatment of *Exoc5* CKO mice with z-VAD-FMK rescued stratification as indicated by E-cadherin (green) and SMA (red) staining (third row). Positive uroplakin-3 (UPK3) (red) staining in z-VAD-FMK-treated *Exoc5* CKO mice indicated successful differentiation of superficial cells (fourth row). Scale bars: 200 μm (first row; kidney), 20 μm (second, third and fourth rows; ureters). (B) H&E staining of E18.5 kidneys from *Exoc5* CKO mice with and without z-VAD-FMK treatment showed subsequent absence of hydronephrosis. Scale bar: 200 μm.

As TWEAK–Fn14 and non-canonical NF-κB signaling can initiate caspase activity and cell death, we explored whether *Exoc5* CKO urothelial death could be prevented *in vivo* with a pan-caspase inhibitor (Ikner and Ashkenazi, 2011; Martin-Sanchez et al., 2018; Vince et al., 2008). To perform this, we administered 5 μg/g z-VAD-FMK by intraperitoneal injection to timed mated female mice at E16.5 and collected embryos at E18.5. Under normal conditions, the *Exoc5* CKO urothelium fails to stratify and undergoes cell death between E16.5 and E17.5, with full ureter obstruction by E18.5 as the underlying mesenchyme obliterates the ureter lumen (Fogelgren et al., 2015; Lee et al., 2016) (Fig. 7A). However, when administering z-VAD-FMK at E16.5, we observed no urothelial cell death and complete rescue of proper ureter formation (*n*=9/9 *Exoc5* CKO embryos) (Fig. 7A). Furthermore, the *Exoc5* CKO urothelium successfully stratified as seen by UPK3 immunofluorescence in the umbrella cells (Fig. 7A). These *in vivo* results agreed with our *ex vivo* explant data and previously published data (Lee et al., 2016), which indicated that obstruction was the subsequent result of widespread urothelial cell death. With the obstruction prevented, we observed that hydronephrosis was avoided in these *Exoc5* CKO embryos as well (Fig. 7B). In addition, although *Exoc5* CKO mice typically died 8-14 h after birth, E16.5 rescue trials revealed that all z-VAD-FMK-treated Exoc5 CKO mice successfully survived to adulthood.

## DISCUSSION

Although defects in membrane trafficking are known to contribute to a breadth of human diseases, it remains unclear whether or not aberrant exocyst function contributes to developmental defects of the urinary tract, such as COU (Coulter et al., 2020; Uhm et al., 2017; Van Bergen et al., 2020). The phenotypic mirroring of human COU by the *Exoc5* CKO mouse offers a congenital model for deconstructing the molecular mechanisms underlying the onset of obstructive uropathy. Nephrogenesis is dependent on ureteric bud branching into the metanephric mesenchyme, which starts around E11.5, and we have previously shown that Cre recombinase driven by the *Ksp*-cadherin promoter is active by this time (Lee et al., 2016). Thus, *Exoc5* is not required for branching morphogenesis by the ureteric bud, and the urinary tract appears morphologically normal until E17.5. However, we observed that *Exoc5* ablation in the urothelium resulted in autophagy impairment and non-canonical NF-κB activity prior to cell death at E17.5, for which we further report TUNEL-positive and cleaved caspase 3-positive signals only from a small pool of cells that had already detached from the ureter wall. These data suggest the loss of cell anchorage in a minority of urothelial cells might play a role in inducing anoikis, which is a form of apoptosis. Taken together, these data support a model in which exocyst depletion promotes impaired autophagy, and that autophagic deficiencies in turn activate progressive NF-κB signaling and eventually cell death. Although the exact series of mechanisms will need to be further investigated, it is clear that the *Exoc5* CKO model of COU can be rescued with the pan-caspase inhibitor z-VAD-FMK.

The relationship between defective autophagy and NF-κB signaling is intricately tied to cellular stress and damage response. For example, deficiencies in autophagy have been shown to activate canonical NF-κB signaling in part through an accumulation of p62, which serves as a scaffold for the TRAF6 and RIP1 complex that drives canonical NF-κB activation (Meng and Cai, 2011; Wooten et al., 2005). Autophagy typically suppresses p62 accumulation, thereby guarding against unnecessary canonical NF-κB signaling (Mathew et al., 2009). In the data presented here, we observed LC3 and p62 accumulation when inhibiting exocyst function, which suggests a path for activating canonical NF-κB signaling as part of a survival response in urothelial cells. More specifically, it is reasonable to posit that inhibiting EXOC5 would further impact the formation of the RALB–EXOC8 effector complex, and this would have a direct impact on the ability to initiate autophagic response to stress. The relationship between autophagy deficiency and canonical NF-κB activation is further supported here by BafA1 treatment-induced RelA nuclear translocation. The initial canonical NF-κB response to BafA1 treatment might be an attempt to promote cell survival in the face of cell stress. However, these data further indicated that sustained stress in urothelial cells promotes a slower second non-canonical NF-κB response.

One way to detect defective autophagy is the accumulation of LC3, as was observed in the *Exoc5* CKO ureter urothelium at E17.5. This LC3 accumulation was enhanced upon *Exoc5* ablation in the adult bladder urothelium in *Upk3a-GCE* mice. The urothelial *Exoc5* ablation presented here in the ureter and bladder highlight a tissue- and time-specific relationship between the exocyst and autophagy. It is likely that *Exoc5* ablation in the bladder urothelium presented with larger LC3 puncta because there was a longer time frame (4 weeks) for this buildup to occur. This observation would also support the hypothesis that an exocyst-mediated event initiated the stress response in the developing ureter and that the impaired autophagy contributed to NF-κB activation.

Interestingly, ablation of *Exoc5* would also impact formation of the RALB–EXOC2 effector complex, which directly activates TBK1. TBK1 plays a critical role in inhibiting cell death and maintaining non-canonical NF-κB signaling by phosphorylating NF-κB-inducing kinase (NIK), which leads to NIK degradation (Chien et al., 2006; Jin et al., 2012). NIK degradation is necessary for preventing p100 processing to p52 (Xiao et al., 2001). By extension, the urothelial stress induced by *Exoc5* ablation could reasonably be expected to impact RALB–EXOC2 effector complex interaction with TBK1 and thereby promote non-canonical NF-κB signaling through NIK accumulation. As the exocyst acts as a moderator of these pathways, it will be necessary in the future to confirm these interactions in ureter development.

Our observation that non-canonical p52 nuclear translocation acts as a slower response complements results reported by Saitoh et al. (2003), in which TWEAK was directly used to stimulate non-canonical NF-κB signaling independently of TNFα in mouse embryonic fibroblasts, and which found that p52 nuclear translocation was delayed in response compared to canonical RelA nuclear translocation (Saitoh et al., 2003). Under these conditions, canonical RelA responded first but was followed by non-canonical p52 after 8 h, whereafter p52 remained in the nucleus for at least 24 h (Saitoh et al., 2003). Depletion of exocyst function and subsequent autophagic deficiency contribute added complexity to the ability of the cells to deal with stress. Progression to TWEAK–Fn14-mediated non-canonical NF-κB activation is intriguing because TWEAK can induce several cell death pathways, including caspase-mediated death; however, to date, there is not yet a strong body of evidence connecting this pathway to anoikis (Chicheportiche et al., 1997; Nakayama et al., 2003). Here, it is important to state that these data do not suggest that Fn14-mediated non-canonical NF-κB signaling is singularly dependent on the effects of autophagic deficiency, nor that this is the sole driving factor behind *Exoc5* CKO ureter cell death. Rather, these data indicate a role for impaired autophagic flux in promoting a sustained TWEAK and Fn14 environment. More significantly, we report that the pan-caspase inhibitor z-VAD-FMK can prevent urothelial cell death and ureter oblation from occurring in the *Exoc5* CKO mouse model.

In summary, these data demonstrate that urothelial cell death is initiated independently of aberrant urothelial stratification and is the underlying pathological driver of COU in the *Exoc5* CKO mouse model. We further implicate the effects of impaired autophagy in response to stress events during ureter development, wherein there is progressive promotion of canonical and then non-canonical NF-κB signaling. These findings might provide insight into the pathological events contributing to human COU.

## MATERIALS AND METHODS

### Animals

All animal procedures and protocols were conducted in accordance with Institutional Animal Care and Use Committee (IACUC) specifications approved by the University of Hawaiʿi Animal and Veterinary Services. B.F.’s IACUC approved protocol is #11-1094 and the University of Hawaiʿi has an Animal Welfare Assurance on file with the Office of Laboratory Animal Welfare (assurance number A3423-01). Mice were housed under standard conditions with a 12-h light cycle with water and food *ad libitum*. The floxed EXOC5 mouse strain (*EXOC5^FL^*) was generated and used as previously described in Fogelgren et al. (2015), as was the *tdTomato* Cre reporter strain (The Jackson Laboratory, 007909). The Ksp-Cre and *Upk3a-GCE* mouse strains were obtained from The Jackson Laboratory (012237 and 015855, respectively). All mice were on a C57/Bl6/J inbred background. For timed matings, females mated with a male were monitored for abdominal bulging beginning 14 days after arranging timed mating. Embryos were dissected in the morning and measured by embryonic body length and staged using Theiler staging criteria to ensure the developmental stage of each embryo. Six- to 8-week-old *Upk3a-GCE* mice were fed tamoxifen-containing chow (Envigo, TD.130860) for 2 weeks and then, after 4 weeks with normal chow, bladders were collected for histology analysis. For rescue experiments, timed mated female mice were intraperitoneally injected at E16.5 with z-VAD-FMK (R&D Systems, FMK001) at 5 μg/g body weight in 10% DMSO in PBS. Embryos were collected 48 h after injection, with caudal torsos collected for histological analysis and DNA isolated for genotyping.

### Histology and immunohistochemistry

Caudal torsos of *Exoc5* knockout and control animals were dissected and fixed in 4% formaldehyde overnight with rocking at 4°C. Samples were embedded in paraffin according to standard protocols and cut into 5 μm sections. Staining and immunohistochemistry procedures were performed as previously reported (Fogelgren et al., 2015). To detect apoptotic cells in formalin-fixed histological sections, DeadEnd Fluorometric TUNEL System (Promega, G3250) was used according to the manufacturer's instructions. The primary antibodies used were as follows: anti-E-cadherin (1:200, Cell Signaling Technology, 3195), anti-SMA (1:800, Millipore Sigma, A2547), anti-uroplakin-3 (1:100, American Research Products, 03-610108), anti-collagen IV (1:200, Abcam, ab6586) and anti-LC3 (1:500, Cell Signaling Technology, 12741). The secondary antibodies Dylight 488 and Dylight 594 (Thermo Fisher Scientific, 35552 and 35560, respectively) were used at a 1:800 dilution. Stained sections were analyzed using a Olympus BX41 fluorescence microscope. LC3 quantification analysis was performed using ImageJ software (National Institutes of Health).

### Co-immunoprecipitation and western blotting

Samples were lysed in co-immunoprecipitation buffer (50 nM Tris-HCl pH 8, 150 mM NaCl, 5 mM EDTA, 0.5% NP- 40, 1 mM dithiothreitol, 20 mM NaF) containing phosphatase and protease inhibitors via mechanical homogenizing and vortexing. Samples were placed in a microcentrifuge and spun at 20,000 ***g*** at 4°C for 30 min. The supernatant was removed and proteins were quantified by Bradford's assay. Protein input for co-immunoprecipitation was 2 mg, and 8 μg of antibody was used per 2 mg of protein input. Protein samples were incubated with an antibody against EXOC4 (Enzo Life Sciences, ADI-VAM-SV016-D) or rat IgG control (Thermo Fisher Scientific, 02-9602) overnight with end-to-end rotation at 4°C. Immune complexes were then pulled down using Protein A/G Magnetic Beads (Thermo Fisher Scientific, 88802), boiled in 2× Laemmli Sample Buffer (Bio-Rad, 1610737) with β-mercaptoethanol. The supernatant was run using SDS-PAGE and transferred to a nitrocellulose membrane using Trans-Blot Turbo Transfer System (Bio-Rad). The membrane was blocked in 5% non-fat milk for 1 h and probed with primary antibody overnight. Secondary antibodies (LI-COR IRDye, 926-32210 and 926-68071) were incubated at 1:10,000 for 1 h followed by three washes in PBS with 0.1% Tween 20 (PBST) washes and scanned on Odyssey CLx Imaging System.

The other antibodies used for western blotting were anti-p62 (1:1000, Cell Signaling Technology, 16177S), anti-ATG7 (1:1000, Cell Signaling Technology, 8558), anti-ATG5 (1:1000, Cell Signaling Technology, 12994), anti-EXOC5 (SEC10) (1:500, Santa Cruz Biotechnology, sc-514802), anti-LC3 (1:1000, Cell Signaling Technology, 12741), anti-Fn14 (EPR3179) (1:1000, Abcam, ab109365) and anti-BACT (1:1000, Cell Signaling Technology, 4970). Image Studio Lite (LI-COR) was used to analyze and pseudocolor all scans to grayscale.

### Cell culture

For immunofluorescence, cells were seeded on coverslips and grown overnight. Treatments with 100 µM endosidin-2 (Cayman, 21888) (Zhang et al., 2016; Fujimoto et al., 2019; Leskova et al., 2020), 100 ng/ml recombinant human TWEAK (R&D Systems, 1090-TW-025/CF), 50 µM VPS34i (Cayman, 17392) or 200 nM BafA1 (Sigma-Aldrich, B1793) were applied in fresh media changes at the indicated time and grown under standard conditions at 37°C and 5% CO_2_ for the respective timepoints listed. Cells were washed three times with PBS followed by fixation for 10 min in 4% paraformaldehyde. Cells were permeabilized for 10 min with 0.1% Triton X-100 in PBS, with three PBST washes occurring between each step. Cells were blocked in 5% BSA in PBST for 1 h, then incubated with the respective primary antibody (1:100) overnight at 4°C. The primary antibodies used were anti-EXOC5 (SEC10), anti-ATG5, anti-p65 (Cell Signaling Technology, 8242S), anti-p52 (Millipore, 05-361), anti-GOL97 (Santa Cruz Biotechnology, sc-59820) and anti-Fn14. Cells were washed thrice in PBST, then incubated with a secondary antibody (Dylight) for 1 h, washed thrice with PBST for 5 min and mounted using VECTASHIELD Antifade Mounting Medium.

### Crystal Violet staining

Approximately 3×10^5^ SV-HUC-1 cells were seeded and transfected with 3 µg MISSON pLKO.1-puro non-mammalian targeting control shRNA (Sigma-Aldrich, SHC002) or shExoc5 (Sigma-Aldrich, TRCN0000061963) using RNAiMAX (Thermo Fisher Scientific, 13778075) according to the manufacturer's protocol. Transfected cells were grown for 72 h followed by puromycin selection. Stable cell lines were confirmed for EXOC5 knockdown by western blotting, plated at 10^5^ cells per well in six-well plates in triplicate and grown for 1 week under vehicle control or 200 nM BafA1 treatment, followed by staining with 0.1% Crystal Violet (CV) solution. CV solution was removed with repeated H_2_O washes and the plate was scanned. The percentage of area coverage was measured using ImageJ software and set relative to control.

### Cell viability assay

Approximately 10^4^ SV-HUC-1 cells were seeded per well in a 96-well opaque plate. Cells were treated and then subsequently assessed for their cell viability by the CellTiter-Glo Luminescent Cell Viability Assay (Promega, G7570). The assay measures ATP as a biomarker of metabolically active cells by the luminescence released from the conversion of Beetle Luciferin+ATP with the enzyme catalyst Ultra-Glo recombinant luciferase and Mg^2+^ to oxyluciferin+AMP+PPi+CO_2_ and light. For the assay, 50 µl of CellTiterGlo reagent and buffer mix was added to 50 µl of cell media in each well and luminescence was measured by SpectraMax M3 (Molecular Devices).

### *Ex vivo* ureter culture model

Timed matings were set up and embryos were collected at gestational age 15.5 (E15.5). Ureters were microdissected and placed on 1.0 μm sterile filters at the air–liquid interface in a 12-well plate in 50/50 Dulbecco's Modified Eagle Medium/Ham's F12 (Thermo Fisher Scientific) supplemented with 5 μg/ml transferrin (Thermo Fisher Scientific), 100 μg/ml penicillin and 100 U/ml streptomycin. The ureters were cultured for 72 h at 37°C. Gross images and peristalsis videos were taken using an Olympus CKX41 microscope. For samples that were treated with chemical agonists or antagonists, media containing the compounds were changed daily, and samples were cultured for 72 h at 37°C. After 72 h in culture, the ureter explants were fixed with 4% paraformaldehyde overnight and subsequently placed in sucrose for cryo-sectioning and histological analysis. Alternatively, the explants were collected for RNA extraction.

### Affymetrix Clariom D GeneChip microarrays

Ureters were microdissected from E16.5 embryos (*n*=3 *Exoc5^FL/FL^* control and *n*=3 *Exoc5* CKOs) and RNA was isolated as previously described (Lee et al., 2016). After confirming RNA quality with an Agilent Bioanalyzer, gene expression profiling was performed at the University of Hawaiʿi Genomics and Bioinformatics Shared Resources using Affymetrix Clariom D gene chips. Transcriptome Analysis Console (TAC) software (Affymetrix) was used to analyze and identify differential expression between wild-type and *Exoc5* CKO samples. GraphPad Prism 9 was used to generate a volcano plot and KEGG analysis was performed with the Database for Annotation, Visualization and Integrated Discovery (DAVID) (Huang et al., 2009; Sherman et al., 2008).

### Statistical analysis

All experiments were performed at least twice in triplicate with the most representative images shown. Error bars are presented as the mean±s.d. and differences between groups were analyzed by two-tailed unpaired Student's *t*-test or one-way ANOVA with Tukey's post hoc test, as indicated. Statistical significance was accepted at *P≤0.05, **P≤0.01, ***P≤0.001 and ****P≤0.0001.

## Supplementary Material

10.1242/dmm.049785_sup1 Supplementary information
