# Supplementary material for: Exocyst inactivation in urothelial cells disrupts autophagy and activates non-canonical NF-κB signaling
Source: Dis Model Mech. 2022 Oct 12;15(10):dmm049785. doi: 10.1242/dmm.049785 (PMC9586569; doi:10.1242/dmm.049785)
Supplement: Supplementary information [file dmm-15-049785-s1.pdf]

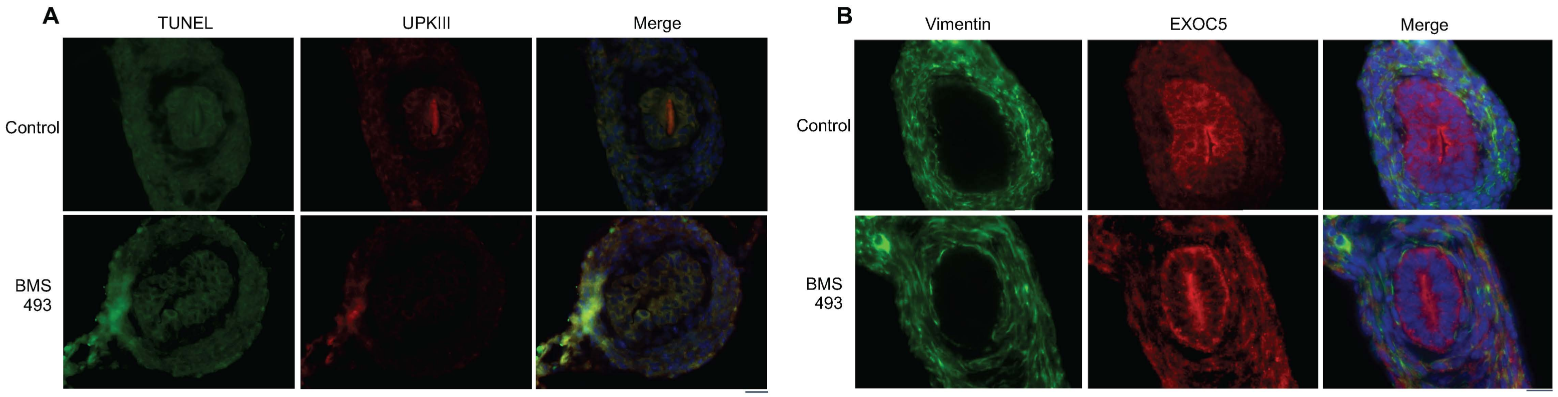


**Fig S1. Immunohistochemistry and EXOC5 immunostaining in *ex vivo* cultured ureter explants treated with BMS493.** (A) Immunohistochemistry of ex vivo cultured ureter explants treated with BMS493 for 72 h revealed no TUNEL-positive urothelial cells, despite inhibition of urothelial differentiation. TUNEL signal is shown in green, and UPK3 staining shown in red. (B) EXOC5 immunostaining in ex vivo cultured ureter explants treated with BMS493. Exoc5 is expressed throughout the urothelial cells with a concentration at the apical luminal membrane. No apparent difference in distribution is observed when urothelial differentiation is blocked with BMS493.

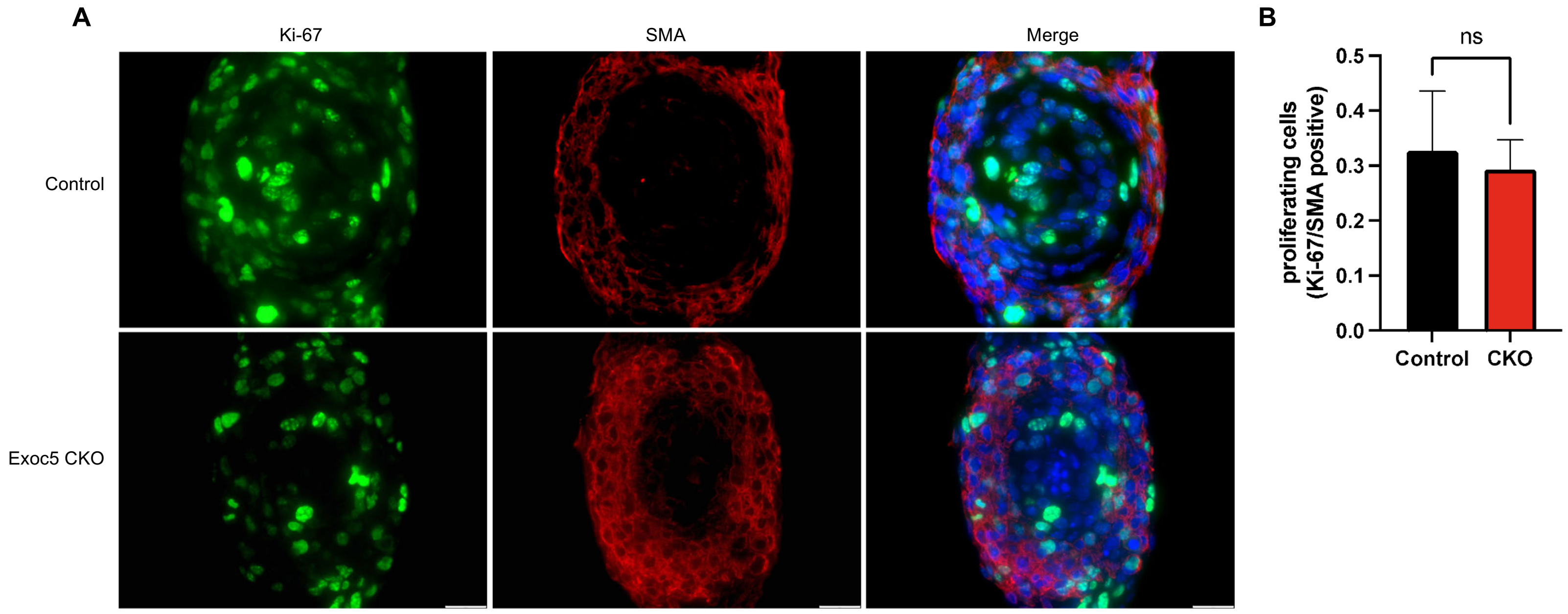


**Fig S2. Immunostaining of Ki67 to measure proliferation rates in *Exoc5* CKO *ex vivo* cultured ureter explants.** (A) Control and *Exoc5* CKO *ex vivo* cultured ureter explants displayed no significant differences in SMA distribution or Ki-67 abundance. (B) Quantitation of Ki-67/SMA show no significant differences after culturing for 72 h, indicating explants did not have the fibroproliferative response that was seen *in vivo* Exoc5 CKO ureters. n.s.= not significant, $P \geq 0.05$.

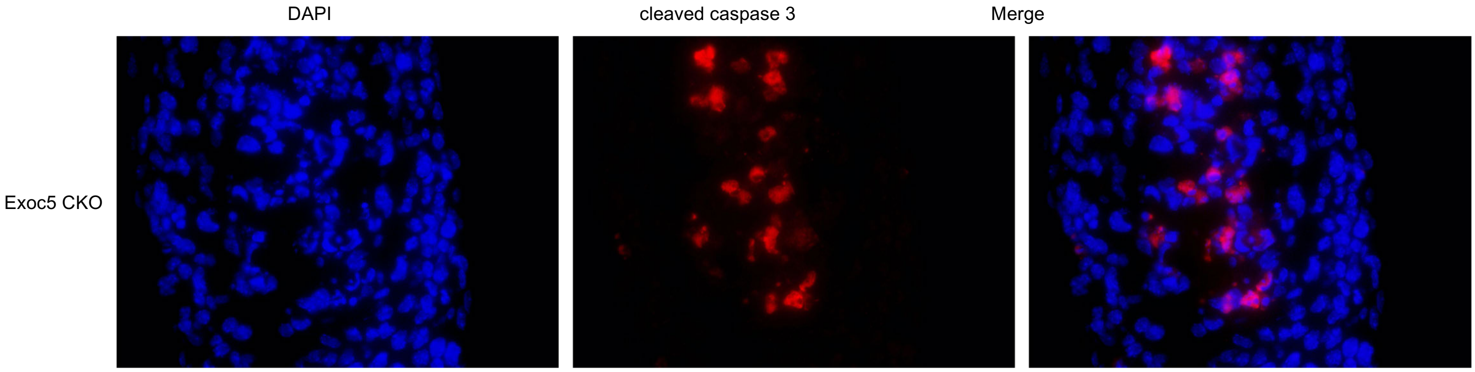


**Fig. S3. Immunostaining of cleaved caspase 3 in *Exoc5* CKO *ex vivo* cultured ureter explants.** Cleaved caspase 3 is detected in a portion of cells that undergo basement membrane detachment and cell sloughing.

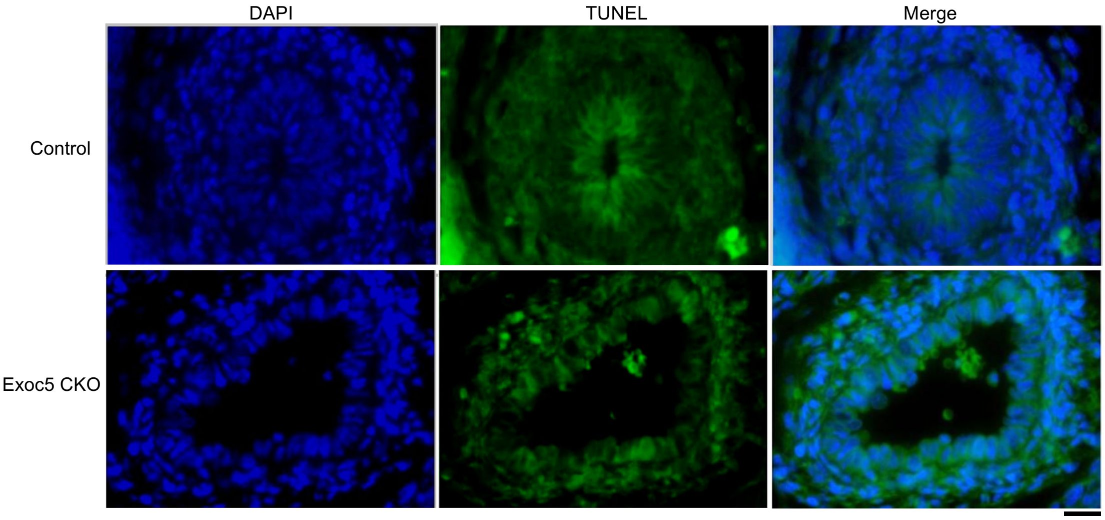


**Fig. S4. TUNEL staining of E17.5 Exoc5 CKO ureters revealed few TUNEL-positive urothelial cells.** Only detached urothelial cells sloughing off into the lumen of E17.5 CKO ureters were TUNEL-positive.

Disease Models & Mechanisms • Supplementary information

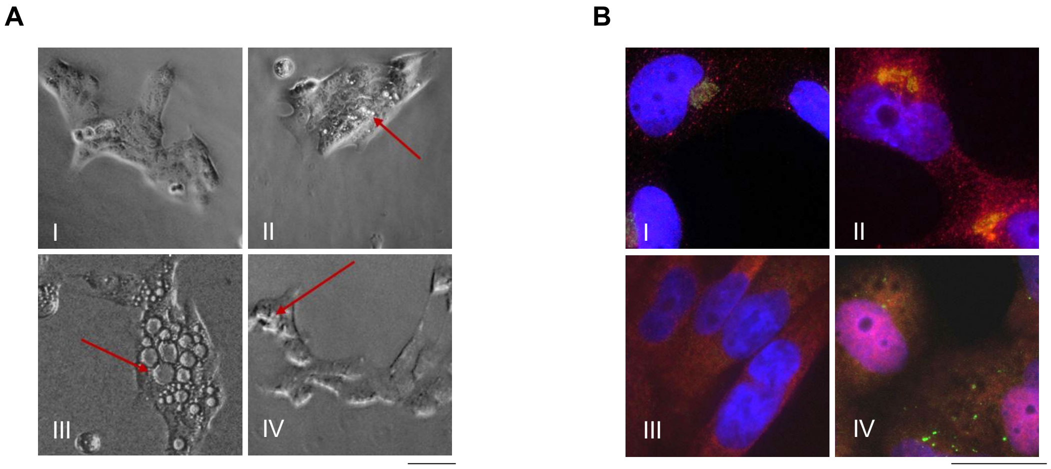


**Fig. S5. Comparative effect of endsidin-2, VPS34i and BafA1 inhibition on SV-HUC-1 cells.** (A) Phase contrast microscopy of SV-HUC-1 cells treated with either (A-I) vehicle control, (A-II) 400 µM Endosidin-2, (A-III) 50 µM VPS34i, or (A-IV) 200 nM BafA1 for 24 h. (B) Immunocytochemistry of SV-HUC-1 cells treated with vehicle control (I, III) or 50 µM VPS34i (II, IV) for 6 h. (B-I,II) Fn14 (red) localization in relation to the Golgi complex as stained by Golgin97 (green). (B-III,IV) Immunohistochemistry of p62 (green) and nuclear translocation of RelA/p65 (red) in response to 50 µM VPS34i treatment. Scale bar=20 µm.

Disease Models & Mechanisms • Supplementary information
